# In vitro studies of the influence of glutamatergic agonists on the Na^+^,K^+^-ATPase and K^+^-*p*-nitrophenylphosphatase activities in the hippocampus and frontal cortex of rats

**DOI:** 10.1186/1477-5751-11-12

**Published:** 2012-05-10

**Authors:** Marcos Brandão Contó, Marco Antonio Campana Venditti

**Affiliations:** 1 Departamento de Psicobiologia, Universidade Federal de São Paulo, Escola Paulista de Medicina (UNIFESP/EPM), Rua Botucatu 862, 1° andar, Vila Clementino, São Paulo, SP, 04023-062, Brazil

**Keywords:** Na^+^,K^+^-ATPase, K^+^-*p*-nitrophenylphosphatase, Glutamate, AMPA, NMDA, Kainate, Frontal cortex, Hippocampus

## Abstract

**Background:**

The overstimulation of excitatory glutamatergic neurotransmission and the inhibition of Na^+^,K^+^-ATPase enzymatic activity have both been implicated in neurotoxicity and are possibly related to the pathogenesis of epilepsy and neurodegenerative disorders. In the present study, we investigated whether glutamatergic stimulation by the glutamatergic agonists glutamate, α-amino-3-hydroxy-5-methyl-isoxazole-4-propionic acid (AMPA), kainate and N-methyl-d-aspartate (NMDA) modulates the Na^+^,K^+^-ATPase and the K^+^-*p*-nitrophenylphosphatase activities in the crude synaptosomal fraction of the hippocampus and the frontal cortex of rats.

**Results:**

Our results demonstrated that these glutamatergic agonists did not influence the activities of Na^+^,K^+^-ATPase or K^+^-*p*-nitrophenylphosphatase in the brain structures analyzed. Assays with lower concentrations of ATP to analyze the preferential activity of the Na^+^,K^+^-ATPase isoform with high affinity for ATP did not show any influence either.

**Conclusions:**

These findings suggest that under our experimental conditions, the stimulation of glutamatergic receptors does not influence the kinetics of the Na^+^,K^+^-ATPase enzyme in the hippocampus and frontal cortex.

## Background

Glutamate is the main excitatory neurotransmitter in the central nervous system [1], and the overstimulation of glutamatergic receptors, including the ionotropic receptors of N-methyl-d-aspartate (NMDA) and α-amino-3-hydroxy-5-methyl-isoxazole-4-propionic acid (AMPA), seems to be related to neuronal death caused by excitotoxicity [1,2]. Glutamate-induced neurotoxicity apparently underlies a variety of neurologic disorders, including epilepsy, Huntington’s disease, Parkinson’s disease and Alzheimer’s disease [1]. Excessive accumulation of glutamate in the synaptic cleft can be due to higher glutamate release, lower uptake by pre-synaptic terminals and/or reverse transportation of glutamate from the pre-synaptic terminal towards the synaptic cleft [2,3]. These processes can result from alterations in the Na^+^ and K^+^ concentration gradients between the intracellular and extracellular environments, which depend on the activity of the transmembrane enzyme Na^+^,K^+^-ATPase [4-6].

Na^+^,K^+^-ATPase is the enzyme responsible for the maintenance of low concentrations of Na^+^ and high concentrations of K^+^ in the intracellular environment, maintaining the resting potential and aiding in the reestablishment of this potential after neuronal depolarization [5]. Structurally, Na^+^,K^+^-ATPase is a heterotrimer formed by three subunits (α_1-4_, β_1-3_ and γ), and the kinetic properties of the isozymes are mainly determined by the α subunit [7]. In the central nervous system, α_1_ and α_2_ function as the “housekeeping” isoforms, while the α_3_ isoform is predominantly activated in situations involving high neuronal activity [8,9]. The inhibition of Na^+^, K^+^-ATPase activity by the glycoside ouabain elicits an excitatory effect leading to convulsions [10,11] and neuronal death through both apoptosis and necrosis [6]. Additionally, a deficiency in Na^+^,K^+^-ATPase activity has been linked to central nervous system disorders, including epilepsy and neurodegenerative diseases [4,6].

Several in vitro studies have been performed in an attempt to understand whether and how glutamatergic activation influences the kinetics of Na^+^,K^+^-ATPase in nervous tissue. The results, however, have not been consistent. Although some researchers have reported a stimulation of Na^+^,K^+^-ATPase enzymatic activity by glutamatergic agonists [12-14], others have demonstrated an inhibition of its activity [15-19] or neither stimulation nor inhibition [20,21]. It is worthwhile to mention that such contradictions may be due to some technical factors, including differences in brain tissues, tissue preparation and the biochemical assays performed; for example, ^42^ K^+^ uptake has been analyzed using scintillation counting [12], Rb^+^ uptake has been analyzed using atomic absorption spectroscopy [19] or scintillation counting [14] and inorganic phosphate release has been analyzed using spectrophotometry [15-17,20,21] and scintillation counting [13]. The results can also be discrepant depending on the family of glutamatergic receptor that is being stimulated [16,18,19].

Therefore, the present study sought to verify whether different concentrations of glutamate and the ionotropic glutamatergic agonists NMDA, AMPA and kainate would alter the kinetic behavior of Na^+^,K^+^-ATPase in the crude synaptosomal fraction of the hippocampus and the frontal cortex. These studies were performed using two different biochemical methods. The first method was a Na^+^,K^+^-ATPase assay involving the complete enzymatic reaction, including both Na^+^-dependent phosphorylation and K^+^-dependent dephosphorylation. The second method was a K^+^*p*-nitrophenylphosphatase (K^+^*p*-NPPase) assay involving only K^+^-dependent dephosphorylation. In the Na^+^,K^+^-ATPase assay, we performed studies with both saturating and sub-saturating concentrations of ATP. Under higher ATP concentrations, the activities of the isozymes containing α_1_, α_2_ and α_3_ are indistinguishable. At lower ATP concentrations, isozymes containing α_3_ can be preferentially assayed because they have a higher affinity for ATP [9]. Concomitant with these assays, we also investigated a putative influence of the glutamatergic agonists on the activities of ouabain-insensitive Mg^2+^-ATPase and Mg^2+^*p*-nitrophenylphosphatase (Mg^2+^*p*-NPPase).

## Results

Tables 1, 2, 3, 4, 5, and 6 show the values (mean ± standard deviation; N = 4-5/group) of K^+^-*p*-NPPase, Mg^2+^-*p*-NPPase, Na^+^,K^+^-ATPase and Mg^2+^-ATPase activities in the frontal cortex and hippocampus, respectively. The analysis of data using the unpaired Student’s *t*-test demonstrated no statistical differences in the activities of K^+^-*p*-NPPase, Mg^2+^-*p*-NPPase, Na^+^,K^+^-ATPase and Mg^2+^-ATPase in the presence (experimental group) or absence (control group) of the glutamatergic agonists utilized. The control and the experimental groups correspond to the same tissue suspensions, and their enzymatic activities were all assayed simultaneously. Five different animals were used for each agonist concentration value (N_TOTAL_: 15 animals)......

**Table 1:** **ATPases and*p*-NPPases activities in the frontal cortex in the presence or absence of 50 μM of glutamatergic agonists**

	**K**^**+**^**-pNPPase** **(10 mM)**	**Mg**^**2+**^**-pNPPase** **(10 mM)**	**Na**^**+**^**,K**^**+**^**-ATPase** **(9 mM)**	**Mg**^**2+**^**-ATPase** ** (9 mM)**	**Na**^**+**^**,K**^**+**^**-ATPase** **(2 mM)**	**Mg**^**2+**^**-ATPase** **(2 mM)**
Control	149.7 ± 15.6	56.48 ± 4.2	280.4 ± 33.8	158.6 ± 22.8	129.5 ± 15.9	109.9 ± 7.4
Glutamate	146.6 ± 25.7	54.83 ± 4.2	299.7 ± 40.9	148.8 ± 26.2	125.6 ± 12.1	107.9 ± 11.2
Control	143 ± 8.5	48.24 ± 3.1	302.8 ± 33.3	139.3 ± 10.7	128.6 ± 25	61.93 ± 5.8
NMDA	148.1 ± 10.3	45.39 ± 3.3	274.7 ± 38.7	148.2 ± 11.8	134.4 ± 26.2	63.45 ± 5.8
Control	131.5 ± 6.5	49.74 ± 2.4	313.9 ± 51.6	178.2 ± 28.8	138.6 ± 24.6	70.42 ± 3.8
AMPA	139.1 ± 6.7	48.64 ± 4.7	325.9 ± 36.7	180 ± 14.3	146.8 ± 20.6	63.26 ± 8.7
Control	134.3 ± 10.5	47.64 ± 5.1	310.7 ± 69.7	147.7 ± 15.2	149.3 ± 13.2	81.58 ± 6.9
Kainate	142.9 ± 15.2	45.4 ± 5.1	319.7 ± 51.9	130.3 ± 13.4	144.6 ± 18.1	83.61 ± 9.8

Values are expressed as mean ± standard deviation (5 animals/group). ATPases activities are expressed in nmol of P_i_/mg protein/min, and *p*-PNPPases activities are expressed in nmol of *p*-nitrophenol/mg protein/min. The concentrations of the substrates are shown in parentheses.

**Table 2:** **ATPases and*p*-NPPases activities in the hippocampus in the presence or absence of 50 μM of glutamatergic agonists**

	**K**^**+**^**-pNPPase** **(10 mM)**	**Mg**^**2+**^**-pNPPase** **(10 mM)**	**Na**^**+**^**,K**^**+**^**-ATPase** **(9 mM)**	**Mg**^**2+**^**-ATPase** **(9 mM)**	**Na**^**+**^**,K**^**+**^**-ATPase** **(2 mM)**	**Mg**^**2+**^**-ATPase** **(2 mM)**
Control	164.3 ± 5.8	28.3 ± 6.5	339.1 ± 89.4	209.5 ± 34.6	198 ± 33.9	99.2 ± 7.4
Glutamate	166.9 ± 8.7	27.2 ± 5.4	355.3 ± 70.6	197.9 ± 36.4	202.8 ± 24.8	91.5 ± 8.9
Control	136.7 ± 13.4	40.8 ± 4.7	463.9 ± 67.1	159.3 ± 22.8	201.2 ± 32.6	94.5 ± 6.7
NMDA	140 ± 12.5	37.4 ± 7.1	432.5 ± 27.7	156.9 ± 38.5	190.4 ± 26.6	94.4 ± 6.5
Control	110.9 ± 12.7	41.5 ± 7.6	407.9 ± 61.9	238.8 ± 41.8	200.6 ± 26.6	79.7 ± 10.3
AMPA	114.4 ± 10.5	38.4 ± 5.6	412.1 ± 74	221.5 ± 42.9	188.3 ± 27.5	71.6 ± 6.3
Control	133 ± 5.2	43.7 ± 7.8	316 ± 119.4	144.9 ± 15.2	205.4 ± 21	83.6 ± 12
Kainate	131.9 ± 11.8	40.3 ± 8	374.7 ± 82.3	124.8 ± 19.7	205.5 ± 22.4	74.5 ± 7.8

Values are expressed as mean ± standard deviation (4–5 animals/group). ATPases activities are expressed in nmol of P_i_/mg protein/min, and *p*-PNPPases activities are expressed in nmol of *p*-nitrophenol/mg protein/min. The concentrations of the substrates are shown in parentheses.

**Table 3:** **ATPases and*p*-NPPases activities in the frontal cortex in the presence or absence of 300 μM of glutamatergic agonists**

	**K**^**+**^**-NPPase** **(10 mM)**	**Mg**^**2+**^**-pNPPase** **(10 mM)**	**Na**^**+**^**,K**^**+**^**-ATPase** **(9 mM)**	**Mg**^**2+**^**-ATPase** **(9 mM)**	**Na**^**+**^**,K**^**+**^**-ATPase** **(2 mM)**	**Mg**^**2+**^**-ATPase** **(2 mM)**
Control	162.2 ± 10	66.4 ± 3.3	303.8 ± 32.4	124.4 ± 13.2	154.0 ± 15.9	129.9 ± 16.1
Glutamate	177.5 ± 15	65.2 ± 5.8	343.6 ± 72.2	104.8 ± 17.2	149.2 ± 10.5	126.8 ± 10.7
Control	214.0 ± 23.2	65.1 ± 3.1	283.5 ± 30.4	192.8 ± 23.2	134.6 ± 15.9	139.3 ± 7.8
NMDA	215.5 ± 16.1	69.5 ± 6.7	270.6 ± 78.9	174.3 ± 31.7	130.7 ± 17.2	131.9 ± 12.5
Control	168.1 ± 13.6	61.6 ± 2.4	407.0 ± 53.4	219.7 ± 21.5	159.5 ± 10.9	128.1 ± 13.6
AMPA	170.1 ± 13.6	61.6 ± 5.4	383.7 ± 79.8	195.4 ± 36	146.3 ± 25	122.7 ± 10.1
Control	162.9 ± 6.9	66.2 ± 3.6	341.0 ± 39.8	192.0 ± 22.4	161.0 ± 17.9	129.0 ± 14.5
Kainate	171.3 ± 9.4	65.8 ± 7.6	346.1 ± 80.3	166.8 ± 16.3	148.8 ± 28.8	127.1 ± 10.5

Values are expressed as mean ± standard deviation (5 animals/group). ATPases activities are expressed in nmol of P_i_/mg protein/min, and *p*-PNPPases activities are expressed in nmol of *p*-nitrophenol/mg protein/min. The concentrations of the substrates are shown in parentheses.

**Table 4:** **ATPases and*p*-NPPases activities in the hippocampus in the presence or absence of 300 μM of glutamatergic agonists**

	**K**^**+**^**-NPPase** **(10 mM)**	**Mg**^**2+**^**-pNPPase** **(10 mM)**	**Na**^**+**^**,K**^**+**^**-ATPase** **(9 mM)**	**Mg**^**2+**^**-ATPase** **(9 mM)**	**Na**^**+**^**,K**^**+**^**-ATPase** **(2 mM)**	**Mg**^**2+**^**-ATPase** **(2 mM)**
Control	155.8 ± 7.4	52.6 ± 1.8	370.7 ± 26.2	205.4 ± 6.5	152.9 ± 13.9	149.7 ± 16.8
Glutamate	153.3 ± 6	56.2 ± 4.9	364.9 ± 32.9	189.4 ± 14.5	166.6 ± 21.5	136.3 ± 11.4
Control	175.0 ± 10.5	56.1 ± 2.4	348.7 ± 45.2	211.6 ± 16.8	156.1 ± 26.4	156.6 ± 13.6
NMDA	176.7 ± 13	57.0 ± 3.3	379.0 ± 21.7	195.8 ± 18.8	143.1 ± 18.5	155.6 ± 18.5
Control	129.7 ± 10.3	57.9 ± 5.8	360.8 ± 24.1	239.6 ± 13.2	146.1 ± 16.5	171.8 ± 23.5
AMPA	142.6 ± 16.3	55.8 ± 3.1	364.9 ± 19.7	230.0 ± 12.5	146.2 ± 12.1	165.5 ± 12.1
Control	189.0 ± 10.5	58.0 ± 3.3	345.6 ± 32	237.0 ± 16.3	180.1 ± 12.5	170.0 ± 22.6
Kainate	184.0 ± 9.8	60.8 ± 2.9	336.9 ± 44.7	221.2 ± 12.9	163.8 ± 22.6	164.3 ± 20.6

Values are expressed as mean ± standard deviation (5 animals/group). ATPases activities are expressed in nmol of P_i_/mg protein/min, and *p*-PNPPases activities are expressed in nmol of *p*-nitrophenol/mg protein/min. The concentrations of the substrates are shown in parentheses.

**Table 5:** **ATPases and*p*-NPPases activities in the frontal cortex in the presence or absence of 1 mM of glutamatergic agonists**

	**K**^**+**^**-pNPPase** **(10 mM)**	**Mg**^**2+**^**-pNPPase** **(10 mM)**	**Na**^**+**^**,K**^**+**^**-ATPase** **(9 mM)**	**Mg**^**2+**^**-ATPase** **(9 mM)**	**Na**^**+**^**,K**^**+**^**-ATPase** **(2 mM)**	**Mg**^**2+**^**-ATPase** **(2 mM)**
Control	169.6 ± 19.4	53.7 ± 3.3	289.4 ± 22.4	138.7 ± 14.7	173.0 ± 31.7	192.8 ± 22.3
Glutamate	167.2 ± 20.3	55.0 ± 3.1	255.2 ± 35.3	129.5 ± 5.8	162.4 ± 6.5	170.5 ± 24.8
Control	169.4 ± 8.9	53.4 ± 3.6	305.7 ± 22.1	159.5 ± 18.5	161.6 ± 10.9	188.4 ± 16.1
NMDA	168.5 ± 3.1	53.8 ± 2.4	275.3 ± 38.9	146.7 ± 7.1	157.5 ± 17.7	176.8 ± 12.3
Control	172.5 ± 11.2	41.7 ± 2.9	265.2 ± 36.4	202.4 ± 12.7	141.4 ± 15.9	165.5 ± 24.3
AMPA	174.2 ± 17.4	41.2 ± 1.8	304.0 ± 21	213.4 ± 17.7	136.7 ± 19.4	167.6 ± 12.1
Control	165.3 ± 18.5	57.6 ± 1.8	296.8 ± 46.9	205.8 ± 17.7	154.6 ± 13.4	166.7 ± 12.5
Kainate	179.9 ± 23.7	55.5 ± 6.9	257.8 ± 35.1	189.6 ± 13.4	149.3 ± 27.7	163.7 ± 11.6

Values are expressed as mean ± standard deviation (5 animals/group). ATPases activities are expressed in nmol of P_i_/mg protein/min, and *p*-PNPPases activities are expressed in nmol of *p*-nitrophenol/mg protein/min. The concentrations of the substrates are shown in parentheses.

**Table 6:** **ATPases and*p*-NPPases activities in the hippocampus in the presence or absence of 1 mM of glutamatergic agonists**

	**K**^**+**^**-pNPPase** **(10 mM)**	**Mg**^**2+**^**-pNPPase** **(10 mM)**	**Na**^**+**^**,K**^**+**^**-ATPase** **(9 mM)**	**Mg**^**2+**^**-ATPase** **(9 mM)**	**Na**^**+**^**,K**^**+**^**-ATPase** **(2 mM)**	**Mg**^**2+**^**-ATPase** **(2 mM)**
Control	140.8 ± 19.4	52.6 ± 4.5	286.6 ± 25.3	205.7 ± 25.9	146.7 ± 14.5	141.7 ± 14.5
Glutamate	143.2 ± 9.8	52.5 ± 3.3	271.2 ± 36	190.0 ± 14.5	136.2 ± 14.7	145.4 ± 18.1
Control	107.2 ± 7.6	54.9 ± 5.8	327.2 ± 22.1	211.8 ± 22.1	137.9 ± 12.1	149.4 ± 10.9
NMDA	109.3 ± 9.2	56.7 ± 3.3	320.3 ± 32.4	204.6 ± 16.1	125.5 ± 10	150.1 ± 4.9
Control	163.1 ± 15.2	49.7 ± 6	300.6 ± 18.5	192.0 ± 28.6	156.2 ± 12.7	153.3 ± 17.7
AMPA	159.5 ± 21.2	50.9 ± 3.3	304.7 ± 15.6	187.3 ± 8.9	155.3 ± 21	150.3 ± 15.4
Control	134.0 ± 11.6	52.5 ± 4.7	290.7 ± 15.6	191.9 ± 20.1	154.2 ± 18.8	149.6 ± 18.1
Kainate	134.8 ± 10.1	54.1 ± 1.6	276 ± 31.7	172.3 ± 13.2	137.3 ± 13	148.6 ± 13.6

Values are expressed as mean ± standard deviation (5 animals/group). ATPases activities are expressed in nmol of P_i_/mg protein/min, and *p*-PNPPases activities are expressed in nmol of *p*-nitrophenol/mg protein/min. The concentrations of the substrates are shown in parentheses.

## Discussion

The literature has elucidated how alterations in Na^+^,K^+^-ATPase activity influence glutamatergic neurotransmission and how both of these elements are correlated to neurotoxicity, possibly contributing to a variety of neurological disorders, including epilepsy and neurodegenerative diseases. The excitatory effect and the apoptotic/necrotic processes elicited by the inhibition of Na^+^,K^+^-ATPase activity in the central nervous system are probably due to several factors: (a) a decrease in the threshold to trigger subsequent action potentials because the accumulation of Na^+^ leads to a lower intracellular electronegativity [6], (b) the increasing release of excitatory neurotransmitters into the synaptic cleft [22,23], (c) a reduction in the pre-synaptic uptake of glutamate from the synaptic cleft, a Na^+^-dependent process [2] and (d) the non-vesicular liberation of glutamate from the intracellular to the extracellular environment through the process of inverse transport by the glutamate transporters [2,3].

Nevertheless, it is still unclear whether and how Na^+^,K^+^-ATPase is influenced by the stimulation of glutamatergic receptors. It has been previously demonstrated that glutamatergic ionotropic agonists stimulate, in a dose-dependent manner, the total activity of Na^+^,K^+^-ATPase in primary cultures of rat cerebral neurons [12]. This result is in agreement with other studies using cultures of cerebellar neurons from rats [13] and in cultures of cerebral cortical astrocytes of mice [14], although these studies only used the neurotransmitter glutamate. However, a decrease in Na^+^,K^+^-ATPase activity after glutamatergic stimulation by NMDA receptor agonists has been observed in slices of rat cerebral cortex [19] and in cerebellar granule cells [16-18]. In another study, a decrease in Na^+^,K^+^-ATPase activity has been observed in rat brain cortex synaptosomes using glutamate [15]. Other studies found neither stimulation nor inhibition of Na^+^,K^+^-ATPase activity in the presence of different concentrations of glutamate in mouse brain synaptosomes [20] or in synaptosomes from the cerebral cortex from rats [21]. The enhancement of Na^+^,K^+^-ATPase activity could be interpreted as a negative feedback mechanism with a protective effect against glutamate neurotoxicity. On the other hand, the decrease in the Na^+^,K^+^-ATPase activity could be interpreted as a positive feedback mechanism that would potentiate glutamate neurotoxicity.

The current work indicated that the stimulation of glutamatergic receptors did not influence the Na^+^,K^+^-ATPase activity in the frontal cortex and hippocampus, as analyzed using the K^+^*p*-NPPase and Na^+^,K^+^-ATPase methods. Assays performed under high concentrations of Na^+^,K^+^ and ATP simulated a situation of high neuronal activity [8,9], such as those elicited by glutamatergic hyperactivity, when the isozymes are indistinguishably activated. On the other hand, assays performed under lower concentrations of ATP simulated a situation of intense and prolonged glutamate-induced depolarization resulting in decreased intracellular ATP concentrations, such as in cases of epileptiform activity [24] and glutamate-induced neurotoxicity [25], and revealed a preferential activity of isozymes containing α_3_. However, we did not find any statistical differences. The lack of differences in our study is in agreement with previous reports in which glutamate did not alter the enzymatic activity in brain synaptosomes of mice [16] and the cerebral cortex of rats [17], although in this last case, the enzymatic reaction was conducted in the presence of a soluble brain fraction containing Na^+^,K^+^-ATPase modulators. In another study that utilized rat cerebral cortex slices, neither kainate nor AMPA changed Na^+^,K^+^-ATPase activity, although NMDA receptor agonists decreased it [14]. The agonists concentrations used in our study (50 μM, 300 μM and 1 mM) are compatible with the previous researches, in which were used glutamatergic agonists between micromolar and milimolar range [12-21]. Thus, it is highly unlikely that the lack of difference in our study is due to the agonists concentrations. It is possible that the age of the animals represents an important factor in the study. Although we performed the enzymatic reaction with brain tissues from adult rats, the studies using the primary cultured cells were derived from rats with only a few days old.

## Conclusions

Therefore, the data obtained in the present work suggest that under the present experimental conditions, the stimulation of glutamatergic receptors does not influence the Na^+^,K^+^-ATPase activity in the frontal cortex and hippocampus. Further in vitro studies need to be performed, under different conditions, using different brain structures, tissue preparations, technical analysis and ages of the animals, in order to reach a better understanding of the relationship between glutamatergic activation and Na^+^,K^+^-ATPase activity.

## Material and methods

### Animals

Subjects were naïve adult male Wistar rats aged three months and weighing approximately 350 g. They were housed in groups of three to four rats per cage (60 x 50 x 22 cm) in a temperature-controlled environment (23 ± 2°C) with a 12:12 h light–dark cycle (lights on from 7:00 a.m. to 7:00 p.m.) with ad lib food and water. This work was approved by our institution’s Ethics Committee on Animal Research (Proc. 0851/10).

### Assays of the enzymatic activities of K^+^-*p*-NPPase and Mg^2+^-*p*-NPPase

The activity of Na^+^,K^+^-ATPase occurs through two fundamental steps, the Na^+^-dependent phosphorylation and the K^+^-dependent dephosphorylation. While the activity of Na^+^,K^+^-ATPase includes both of these steps, which result in the hydrolysis of the natural substrate ATP into ADP and inorganic phosphate (P_i_), the activity of K^+^-*p*-NPPase consists of the hydrolysis of the artificial substrate *p*-nitrophenylphosphate into *p*-nitrophenol and P_i_ and corresponds to K^+^-dependent dephosphorylation by Na^+^,K^+^-ATPase.

The animals were sacrificed by decapitation. The brains were carefully removed and washed with ice-cold 0.9% (w/v) saline solution, and the frontal cortex and hippocampus were rapidly dissected on a cooled petri dish on crushed ice. After weighing, these structures were stored at −20°C until the preparation of homogenates. The K^+^*p*-nitrophenylphosphatase assay was performed as previously described [26]. The homogenates of the brain structures were prepared in ice-cold 0.32 M sucrose (pH 7.0) (2.5% w/v) using a glass homogenizer tube and a motor-driven Teflon pestle. The homogenates were centrifuged at 900 x g for 10 min at 4°C, and the resulting supernatants were centrifuged at 23,000 x g for 20 min at 4°C. The supernatants were discarded, and the pellets were resuspended in 50 mM Tris/HCl (pH 7.4) containing 1 mM EDTA and centrifuged at 23,000 x g for 20 min at 4°C. The supernatants were discarded, and the pellets were resuspended in 50 mM Tris/HCl (pH 7.4) containing 1 mM EDTA. In small glass test tubes, the total *p*-nitrophenylphosphatase activity was assayed in an incubation medium consisting of 40–60 μg protein of the homogenate, 50 mM Tris/HCl (pH 7.4), 5 mM MgCl_2_, 10 mM *p*-nitrophenylphosphate and 10 mM KCl in the presence or absence of glutamate (Sigma®), kainate (Sigma®), AMPA (Sigma®) or NMDA (Sigma®) (50 μM, 300 μM or 1 mM), resulting in a final volume of 100 μL. The ouabain-insensitive Mg^2+^*p*-NPPase activity was determined in a similar medium that was deficient in K^+^. The reactions were incubated in a shaking water bath for 10 min at 37°C. The reaction was stopped by adding 100 μL trichloroacetic acid (10% w/v), the test tubes were centrifuged, and a 100-μL aliquot of the cleared supernatant was transferred into a test tube containing 600 μL of 1 M Tris-base solution. The samples were read in a spectrophotometer at a wavelength of 410 nm. K^+^*p*-NPPase activity was calculated by subtracting the reaction in the absence of KCl from the reaction in presence of KCl. A standard curve was calculated using six distinct amounts of *p*-nitrophenol ranging from 10–120 nmol. The protein in the homogenates was quantified using bovine serum albumin as a standard [27]. The final results are expressed as nmol of *p*-nitrophenol released/mg protein/min.

### Assays of the enzymatic activities of Na^+^,K^+^-ATPase and Mg^2+^-ATPase

The Na^+^,K^+^-ATPase assays were performed as previously described [28], with some modifications. The homogenates of the brain structures were the same as those used for the K^+^*p*-nitrophenylphosphatase assays. In small glass test tubes, the total ATPase activity was assayed in an incubation medium consisting of 8–12 μg protein of the homogenate, 150 mM Tris/HCl (pH 7.4), 4 mM MgCl_2_, 9 mM (or 2 mM) ATP, 70 mM NaCl and 40 mM KCl in the presence or absence of glutamate, kainate, AMPA or NMDA (50 μM, 300 μM or 1 mM), resulting in a final volume of 90 μL. The ouabain-insensitive Mg^2+^-ATPase activity was determined in a similar medium that was deficient in Na^+^ and K^+^ but contained 1 mM ouabain. The reactions were incubated in a shaking water bath for 30 min at 37°C. The reaction was stopped by adding 20 μL trichloroacetic acid (30% w/v), the test tubes were centrifuged, and a 50 μL aliquot of the cleared supernatant was used for determination of released orthophosphate [29]. The samples were read in a spectrophotometer at a wavelength of 820 nm. Na^+^,K^+^-ATPase activity was calculated by subtracting the reaction in the absence of NaCl and KCl and containing ouabain from the reaction in the presence of NaCl and KCl and lacking ouabain. A standard curve was calculated using three distinct amounts of Na_2_HPO_4_ ranging from 25–100 nmol (Additional files 1,2,3,4 , and 5). The protein in the homogenates was quantified using bovine serum albumin as a standard [27]. The final results are expressed as nmol of P_i_ released/mg protein/min.

### Statistical analysis

The unpaired Student’s t-test was used to compare the activities of Na^+^,K^+^-ATPase, K^+^-*p*-NPPase, Mg^2+^-*p*-NPPase and Mg^2+^-ATPase in the presence and absence of the glutamatergic agonists. The significance level was set at p ≤ 0.05 for all statistical tests. The statistical analyses were performed using Statistica version 6.1. and Prism 3.0.

## Competing interests

The authors declare that they have no competing interest.

## Authors’ contributions

MBC carried out the biochemical assays, performed the statistical analysis, participated in the design of the study and drafted the manuscript. MACV participated in the experimental design and in the conceiving of the study. The authors read and approved the final manuscript.

## Supplementary Material

Additional file 1 **Standard curve depicting the linear relationship between absorbance and amounts of P**_**i**_**(25, 50 and 100 nmoles).** This interval comprises the amount of P_i_ released in our enzymatic assay. Click here for file

Additional file 2 **Standard curve depicting the linear relationship between absorbance and amounts of P**_**i**_**(25, 50 and 100 nmoles).** This interval comprises the amount of P_i_ released in our enzymatic assay. Click here for file

Additional file 3 **Standard curve depicting the linear relationship between absorbance and amounts of P**_**i**_**(25, 50 and 100 nmoles).** This interval comprises the amount of P_i_ released in our enzymatic assay. Click here for file

Additional file 4 **Standard curve depicting the linear relationship between absorbance and amounts of P**_**i**_**(25, 50 and 100 nmoles).** This interval comprises the amount of P_i_ released in our enzymatic assay. Click here for file

Additional file 5 **Standard curve depicting the linear relationship between absorbance and amounts of P**_**i**_**(25, 50 and 100 nmoles).** This interval comprises the amount of P_i_ released in our enzymatic assay. Click here for file
