# Supplementary figures and images for: In vitro studies of the influence of glutamatergic agonists on the Na+,K+-ATPase and K+-p-nitrophenylphosphatase activities in the hippocampus and frontal cortex of rats
Source: J Negat Results Biomed. 2012 May 10;11:12. doi: 10.1186/1477-5751-11-12 (PMC3485154; doi:10.1186/1477-5751-11-12)

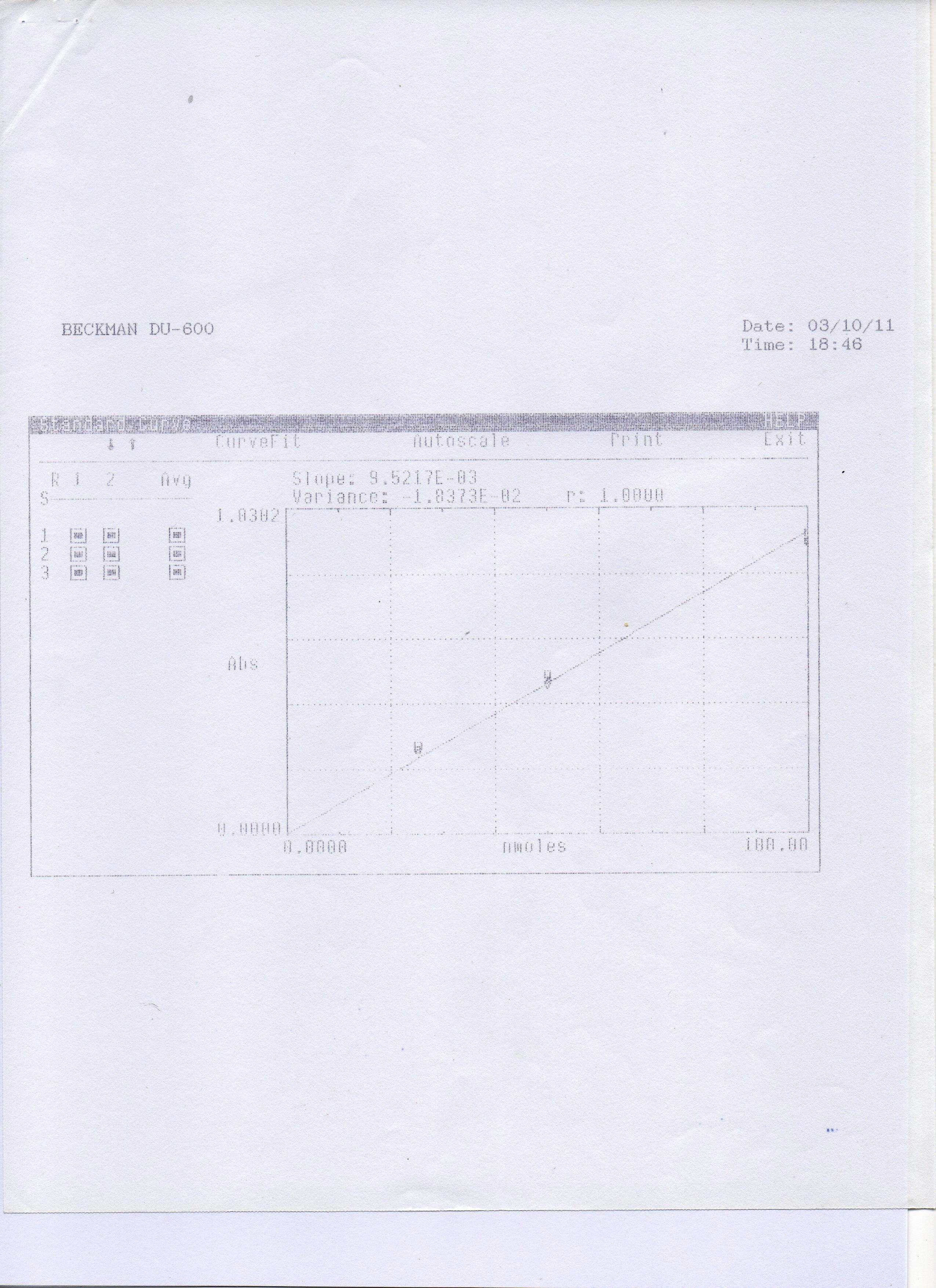

Supplement: Additional file 1 — Standard curve depicting the linear relationship between absorbance and amounts of Pi(25, 50 and 100 nmoles). This interval comprises the amount of Pi released in our enzymatic assay. [file 1477-5751-11-12-S1.jpeg]

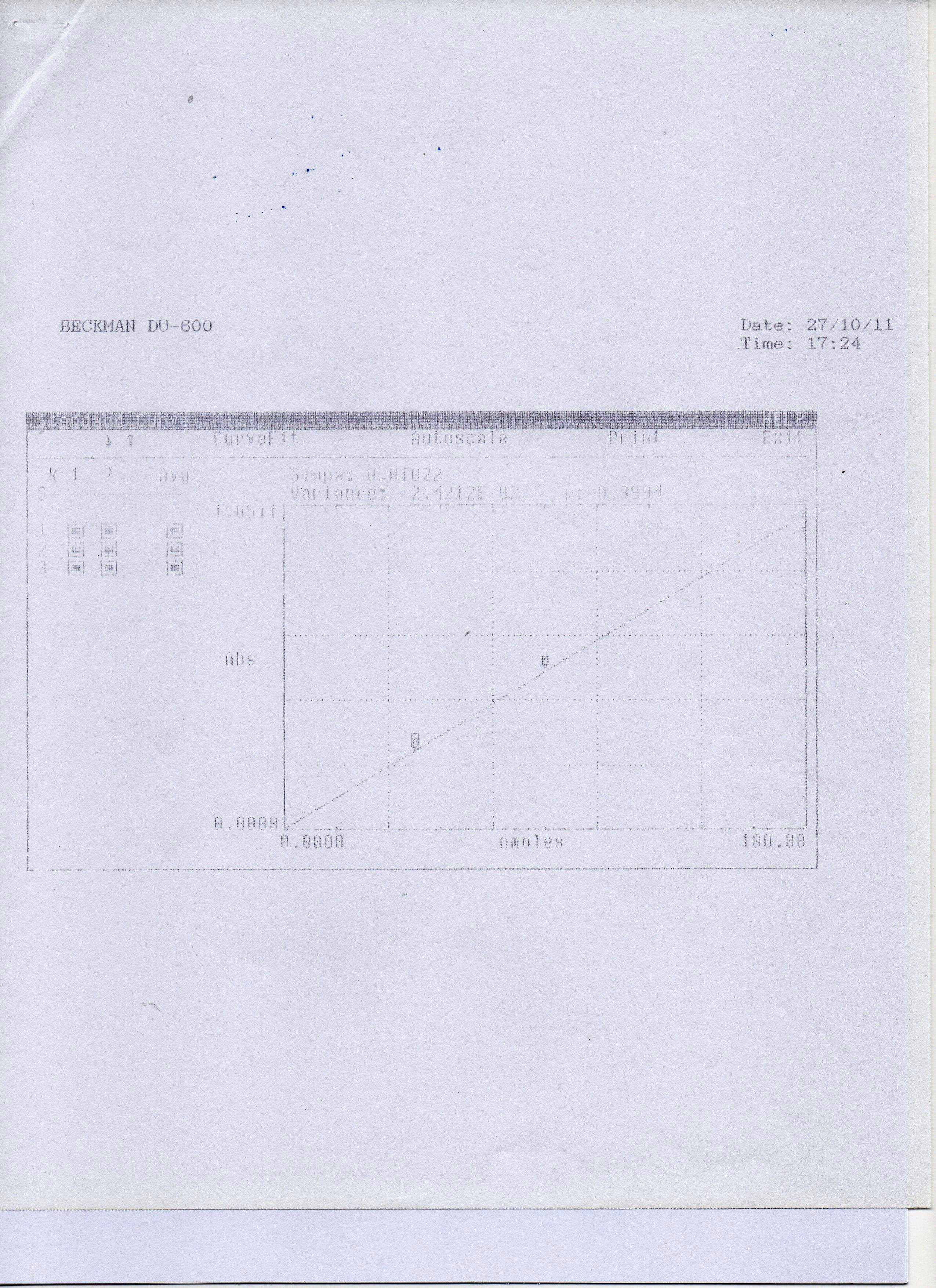

Supplement: Additional file 2 — Standard curve depicting the linear relationship between absorbance and amounts of Pi(25, 50 and 100 nmoles). This interval comprises the amount of Pi released in our enzymatic assay. [file 1477-5751-11-12-S2.jpeg]

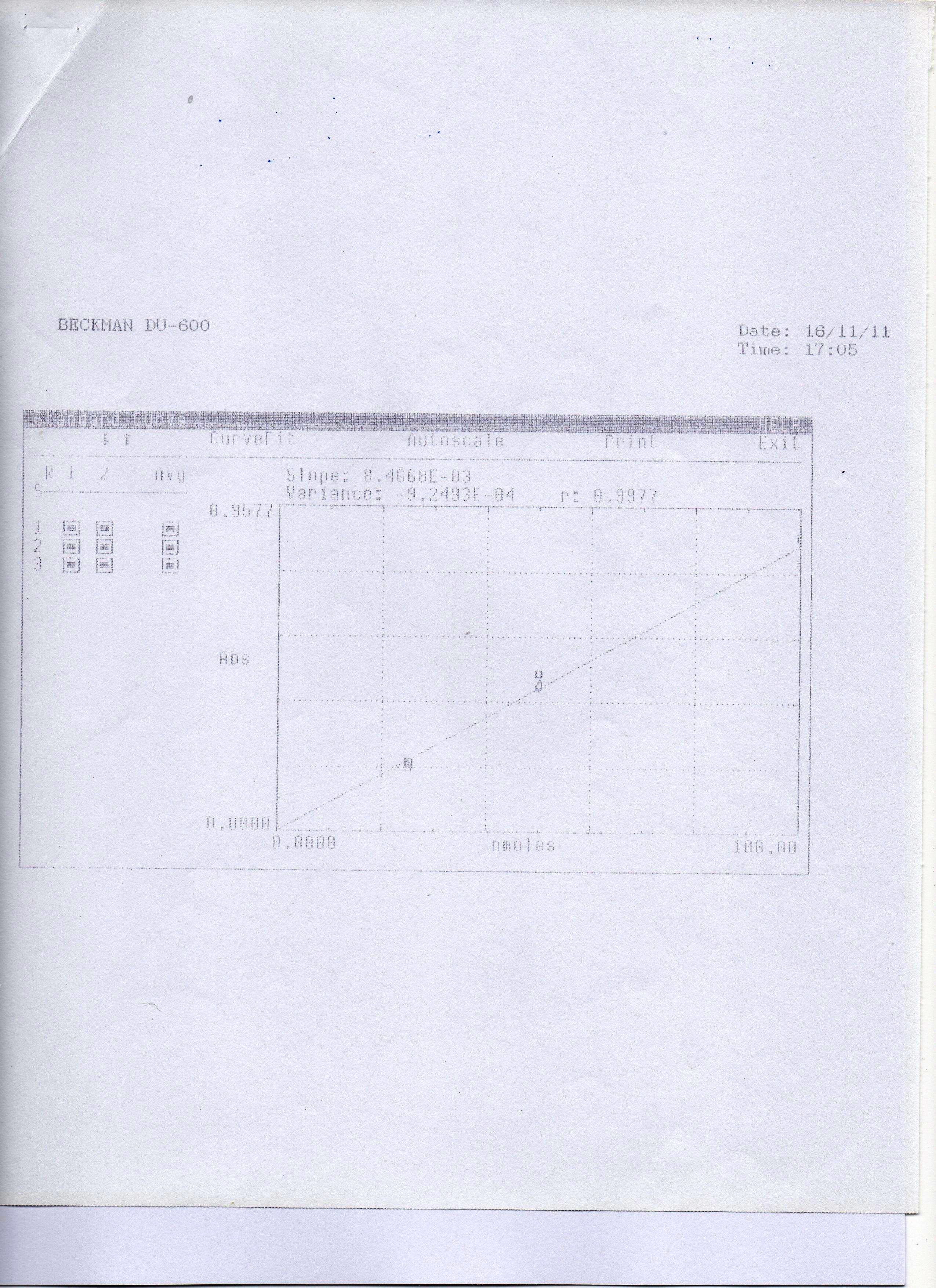

Supplement: Additional file 3 — Standard curve depicting the linear relationship between absorbance and amounts of Pi(25, 50 and 100 nmoles). This interval comprises the amount of Pi released in our enzymatic assay. [file 1477-5751-11-12-S3.jpeg]

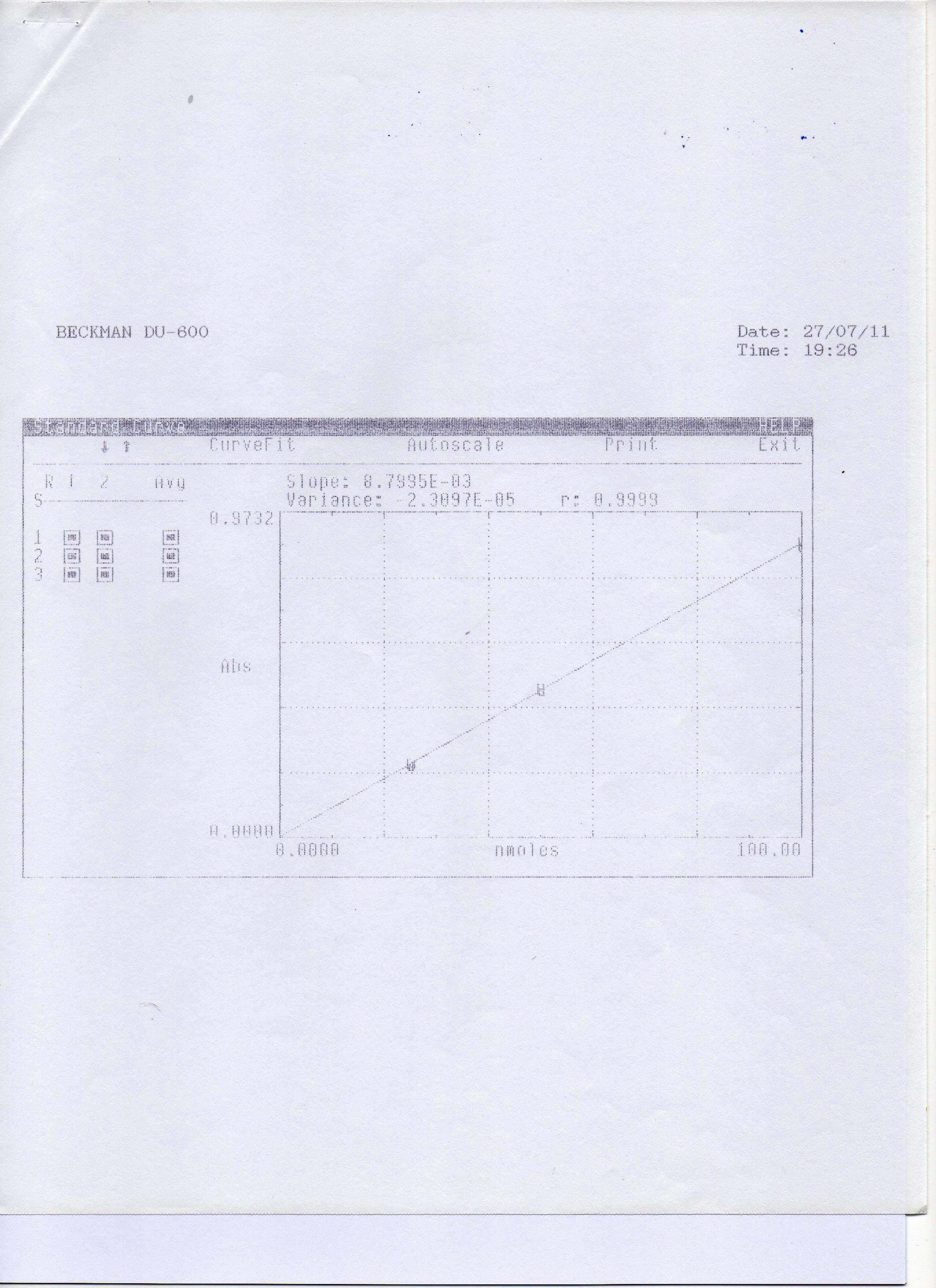

Supplement: Additional file 4 — Standard curve depicting the linear relationship between absorbance and amounts of Pi(25, 50 and 100 nmoles). This interval comprises the amount of Pi released in our enzymatic assay. [file 1477-5751-11-12-S4.jpeg]

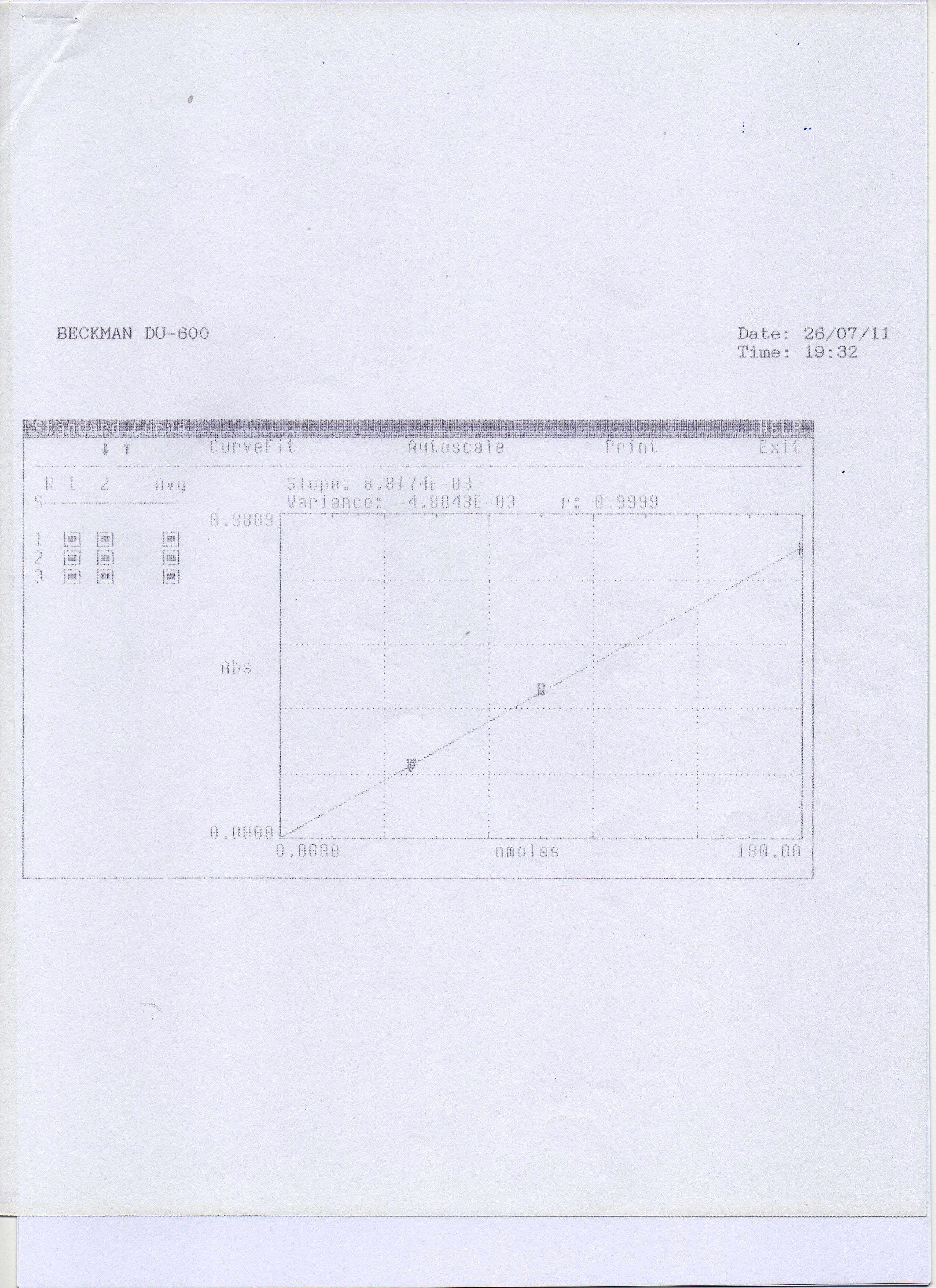

Supplement: Additional file 5 — Standard curve depicting the linear relationship between absorbance and amounts of Pi(25, 50 and 100 nmoles). This interval comprises the amount of Pi released in our enzymatic assay. [file 1477-5751-11-12-S5.jpeg]
